# Cross-Modal Prediction in Speech Perception

**DOI:** 10.1371/journal.pone.0025198

**Published:** 2011-10-05

**Authors:** Carolina Sánchez-García, Agnès Alsius, James T. Enns, Salvador Soto-Faraco

**Affiliations:** 1 Departament de Tecnologies de la Informació i les Comunicacions, Universitat Pompeu Fabra, Barcelona, Spain; 2 Department of Psychology, Queen's University, Kingston, Canada; 3 Department of Psychology, University of British Columbia, Vancouver, Canada; 4 Institució Catalana de Recerca i Estudis Avançats (ICREA), Barcelona, Spain; Max Planck Institute for Human Cognitive and Brain Sciences, Germany

## Abstract

Speech perception often benefits from vision of the speaker's lip movements when they are available. One potential mechanism underlying this reported gain in perception arising from audio-visual integration is on-line prediction. In this study we address whether the preceding speech context in a single modality can improve audiovisual processing and whether this improvement is based on on-line information-transfer across sensory modalities. In the experiments presented here, during each trial, a speech fragment (context) presented in a single sensory modality (voice or lips) was immediately continued by an audiovisual target fragment. Participants made speeded judgments about whether voice and lips were in agreement in the target fragment. The leading single sensory context and the subsequent audiovisual target fragment could be continuous in either one modality only, both (context in one modality continues into both modalities in the target fragment) or neither modalities (i.e., discontinuous). The results showed quicker audiovisual matching responses when context was continuous with the target within either the visual or auditory channel (Experiment 1). Critically, prior visual context also provided an advantage when it was cross-modally continuous (with the auditory channel in the target), but auditory to visual cross-modal continuity resulted in no advantage (Experiment 2). This suggests that visual speech information can provide an on-line benefit for processing the upcoming auditory input through the use of predictive mechanisms. We hypothesize that this benefit is expressed at an early level of speech analysis.

## Introduction

Perceptual information from different sensory systems is often combined to achieve a robust representation of events in the external world [1]. Research during the past two decades has documented numerous instances of multisensory interactions at neuronal and behavioral levels (see [2]). These interactions are demonstrated, for example, in the McGurk effect, such that listening to the spoken syllable /ba/ while simultaneously watching the lip movements corresponding to the syllable /ga/ often results in the illusory perception of /da/ [3]. When visual and acoustic speech signals are correlated, the benefits of multisensory integration in speech perception are also well documented (e.g., [4], [5]). This multisensory advantage is strongest at moderate to high acoustic noise levels [5], [6], when the message is semantically complex [6], [7], or when it involves processing second language sounds [8]. However, the mechanisms that enable this cross-modal benefit are still not well understood.

We hypothesize that one mechanism that could potentially contribute to multisensory speech enhancement is that of predictive coding, operating both within each sensory modality and possibly even between modalities. The principle of predictive coding has been successfully applied, with some variations, to explain information processing in many domains (e.g., [9], [10], [11], [12]), including motor control [13], object identification [14], shape perception [15], [16], music perception [17], visual masking [18], visual search [19], visual spatial orienting [20], [21], and speech perception [22], [23], [24], [25], [26]. What all these proposals have in common is the idea that information in the brain not only flows forward through a hierarchy of processing levels, but that at some stage/s of processing it is also met by a top-down ‘prediction’, projected back from higher levels in the functional hierarchy. These feedback predictions help to reduce ambiguity among potential interpretations of sensory input and to provide finer spatial and temporal parsing of the incoming signals.

In the case of speech, there are several levels of linguistic analysis where on-line predictions might contribute to parse the signal, including phonology, lexical access, syntactic parsing, and semantics. For instance, when listening to a sentence like “I went to a library and borrowed a …”, the expectation to hear “book” is strongly driven by a semantic prior context, but it is likely to constrain lower levels of input analysis including that of phonology and the lexicon (i.e., a strong expectation to hear the phoneme /b/, from the word *book*). Supporting evidence for this has been reported in spoken [27] and written language perception [28], [29]. In Van Berkum's as in DeLong's study, increases in the amplitude of the N400 ERP component were evoked by words that were grammatically incongruent with the most likely continuation in a contextually biasing sentence, even though the remainder of the sentence was never presented. For example, in DeLong et al., the sentence fragment (i.e., “The boy went out to fly …”) could continue with the article *a* (as in “…a kite”, the most likely continuation) or with *an* (as in “… an airplane”, an unlikely continuation). The finding that the unlikely article produced the largest N400 effect was interpreted as evidence for on-line predictions guiding visual (written) word recognition. Furthermore, these predictions seemed to express at the phonological level, because the grammatical, syntactic and semantic aspects of the two possible realizations of the indefinite article were, otherwise, equivalent.

An important question that still remains unexplored is whether the predictions made during speech perception can cross from one sensory modality to the other. If so, such predictions may occur at phonological or even pre-phonological levels of processing. For instance, phonology has been proposed as a common representational code for various aspects of speech perception (visual and acoustic) as well as production [22], [24], [30], [31], [32], [33]. Some evidence for a link between auditory and visual speech representations comes from Rosenblum et al. [31], who exposed participants, previously inexperienced in lip reading, to silent video-clips of an actor producing speech. In a subsequent task, the same participants performed auditory word recognition in noise, being more accurate when the words were spoken by the same speaker they had previously experienced visually (but not heard). Another example of cross-modal transfer in speech comes from Kamachi et al. [33], who reported that people are able to match the identity of speakers across face and voice (i.e. cross-modally), according to the authors based on the link between perception and production of speech.

Results such as these demonstrate the potential for cross modal transfer of information in speech perception. The basis for such transfer during off-line tasks could be phonological or pre-phonological, given the putative relation at these early representation levels between speech perception and production. However, what has not been established to date is a clear demonstration that such transfer is possible in an on-line task, akin the type of processing engaged during normal speech perception. Some hints about this possibility do, however, exist. For example, indirect support for on-line transfer can be drawn from the finding that facial articulatory movements typically precede (and strongly correlate with) the corresponding acoustic signal. The lead time of the facial movements over the corresponding sound is on the order of a few tenths to a few hundredths of milliseconds (e.g., [34]). Further indirect support comes from Van Wassenhove et al. [26], who reported a significant speed up of the ERP components N1 and P2 when they were evoked by audiovisual syllable presentations as compared to audio presentations alone. Interestingly, the size of this latency shift in the auditory evoked components was proportional to the visual saliency of the phoneme, but no correlate of a behavioral benefit was tested. These cross-modal effects on ERP latency, may not necessarily be based on speech-specific mechanisms, as shown by Stekelenburg and Vroomen [35], but abide to a more general mechanism from which speech processing can capitalize.

**Figure 1:**
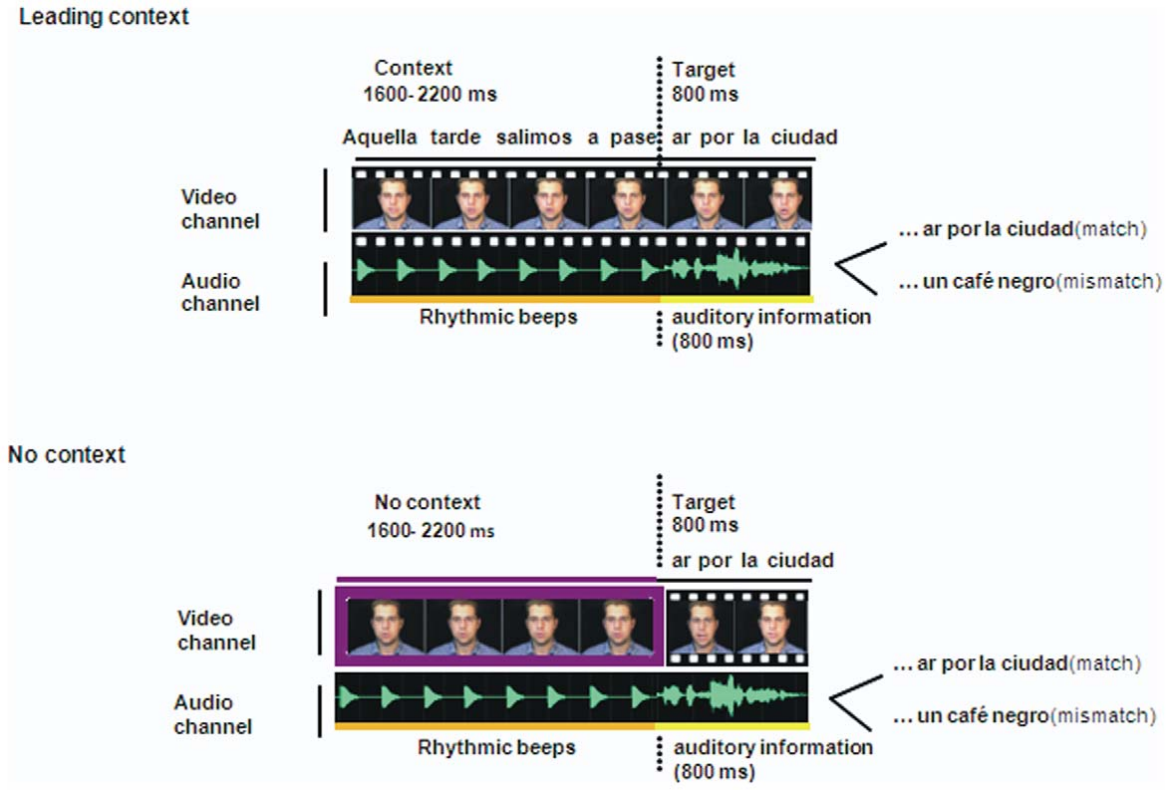
Illustration of the stimulus sequences in Experiment 1. In the example is shown the visual version of the experiment. For the leading context condition, a video clip of the moving lips of the speaker, presented in conjunction with rhythmic beeps, preceded the combined audio and visual target of the sentence. In the no context condition, the leading context consisted of the still video frame of the speaker and rhythmic beeps. In the auditory version (not shown here), the context in the leading context condition consisted of a still video frame and the original audio channel of the spoken sentence. The no context condition was exactly the same to the one shown in the figure for the visual version. English translation of the sentences: That afternoon we went out to walk… around the town/ a black coffee.

The present study was conducted in an effort to test for possible on-line cross-modal benefits during speech perception. In Experiment 1 we began by asking whether performance in an audiovisual matching task would benefit from prior unimodal contextual information (speech fragment in one sensory modality) that was continuous with one of the channels in the audiovisual target clip. As indicated in Figure 1, participants made speeded responses during the presentation of the target clip, to whether or not the speaker's face talked in agreement with the concurrent auditory stream. The critical manipulation was whether a preceding unimodal sentence context (auditory or visual) was continuous with the target clip or whether no such context was provided. When we found that the context provided a benefit in this task, we were ready, in Experiment 2, to compare the benefits of a sentence context that was continuous within a single sensory channel to a context that was continuous across sensory channels. This manipulation allowed us to directly compare potential benefits of on-line predictions unimodally and cross-modally, again testing in both directions, from vision to audition and vice-versa.

## Results

### Experiment 1: Benefits of prior visual and auditory information

**Figure 2:**
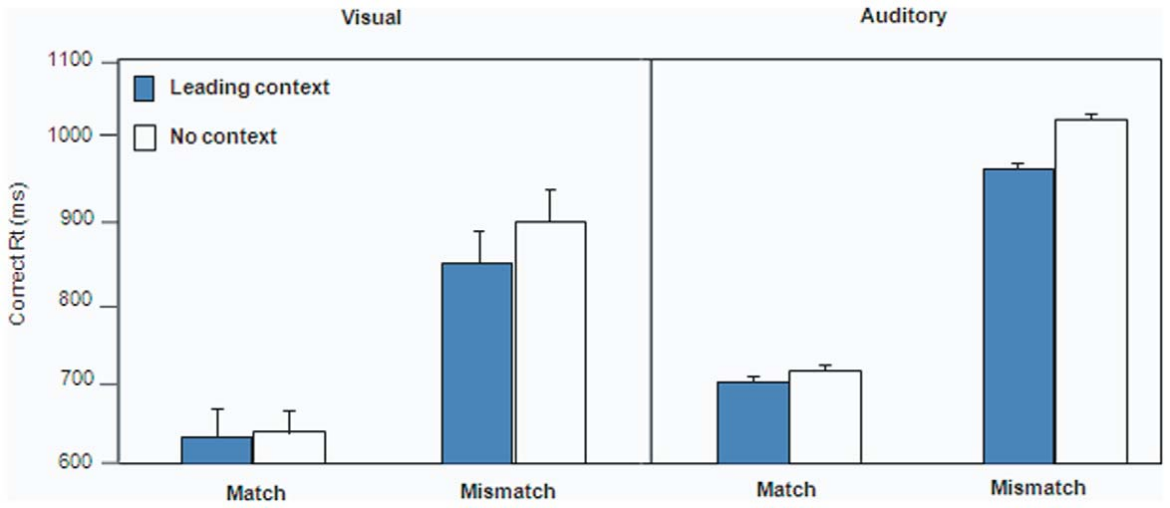
Mean correct RT (in milliseconds) in Experiment 1. Visual (left panel) and auditory (right panel) versions. Error bars represent one standard error of the mean.

We included four types of trials, depending on the information content of the context (unimodal speech or no speech) and the matching nature of the target (audiovisual matching or mismatching). In this experiment, when available, the context was always continuous with the corresponding modality channel in the target fragment. In the auditory version of the experiment, the informative context was auditory, and in the visual version the context was given visually alone. In both cases, the context in the baseline trials (no informative context) contained no speech information. Figure 2 shows the mean correct response times in Experiment 1. In both the visual and the auditory versions, participants detected audiovisual mismatch in the target more rapidly following a leading informative context than no context. This supports the hypothesis that on-line speech perception benefits from advance information in both the visual and auditory modality.

An ANOVA of correct RTs (filtered 2SDs above and below the mean for each participant and condition) indicated faster responses following leading informative context as compared to no context (visual: F(1,15) = 10.42, p<0.05; auditory: F(1,17) = 13.8, p<0.05) and faster responses to matching audiovisual targets than to mismatching ones (visual: F(1,15) = 114.5, p<0.05; auditory: F(1,17) = 368.9, p<0.05). In general, participants were always faster responding after a visual leading context than after an auditory context (t(32) = 2.33; p<0.05). A significant interaction between presence of informative context and target congruency (visual: F(1,15) = 17.8, p<0.05; auditory: F(1,17) = 9.25, p<0.05), reflected that the benefit of context was significant for mismatch trials (visual: t(15) = 5.60, p<0.05; auditory: t(17) = 4.33, p<0.05), but not for match trials (visual: t(15) = 0.44, p = 0.66; auditory: t(17) = 1.18; p = 0.25).

Accuracy was high overall (visual = 88%, auditory = 90%), and did not reflect speed-accuracy trade-offs. We analysed the signal detection parameter *d′* (hits = match responses on matching trials; false alarms = match responses on mismatching trials) and the *criterion*, *C*, as a measure for response bias. In the auditory version, *d′* was higher in presence of leading context (d′ = 2.99 vs. 2.64; t(17) = 3.28; p<0.05), in keeping with the RT pattern. No differences in sensitivity were found in the visual version (d′ = 2.57 vs. 2.67; t(15) = 0.91; p = 0.37). In terms of criterion, both the auditory and the visual versions revealed a stronger bias towards a matching response in the informative context condition as compared to the no context one (auditory, *C* = −0.38 vs. −0.20, t(17) = −3.76; p<0.05; visual, *C* = −0.37 vs. −0.05, t(15) = −4.95; p<0.01).

Experiment 1 provided evidence that audiovisual processing can benefit from information present a few hundred milliseconds earlier in either a visual or an auditory channel. This can reflect the consequences of forming on-line predictions in a cross-modal speech perception task. However, from this result alone one cannot tell whether the leading channel benefits the perception of subsequent speech in the same sensory modality as the leading context, or whether the information in the leading channel can be used to constrain processing in the other sensory modality as well. Experiment 2 was designed to isolate potential cross-modal effects.

### Experiment 2: Cross-modal vs. intra-modal predictions

**Figure 3:**
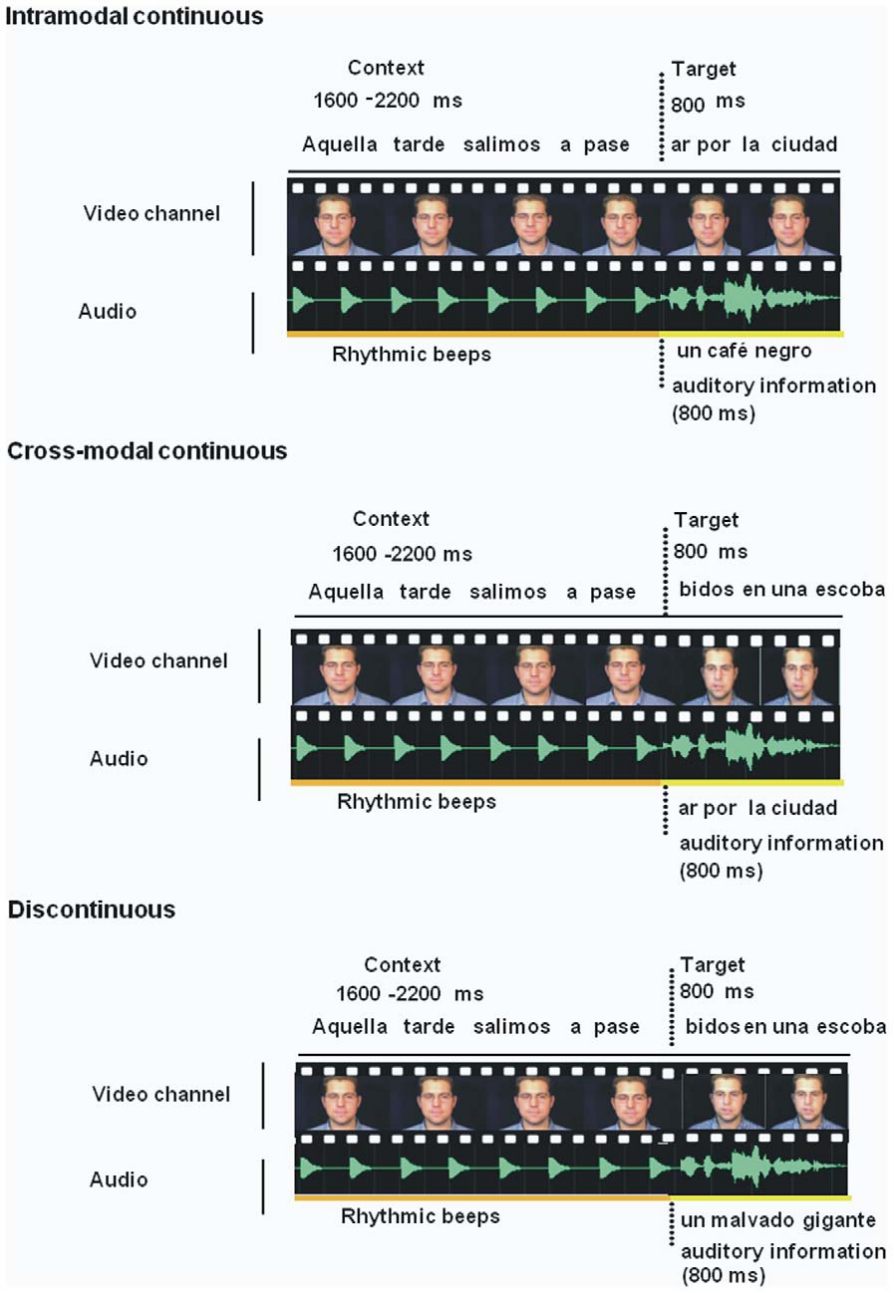
Illustration of mismatch stimulus sequences for the visual version of Experiment 2. In the example shown, for the intra-modal continuous mismatch condition, the lip movements of the context and target fragment were a continuation of the same sentence, but there was no prior information in the auditory channel (rhythmic beeps). In the cross-modal continuous mismatch condition, the lip movements of the context were continuous with the auditory channel of the target fragment. Finally, in the discontinuous mismatch condition, the lip movements of the context and target fragment corresponded to a different sentence. English translation of the sentences: That afternoon we went out to walk… around the town/ a black coffee/ riding a broomstick/ a wicked giant.

This experiment also had visual and auditory versions, each including three main types of trials (see Figure 3). *Intra-modal continuous* (akin to the informative context condition of Experiment 1, where the context continued onto the same sensory modality in the target); *cross-modal continuous* (where the context fragment was continuous only with the opposite sensory modality in the target), and *discontinuous* (where there was no continuity from context to target). In this experiment all trials contained speech information in the context. The intra-modal continuous and the discontinuous trials could have audio-visually matching or mismatching target fragments, but the cross-modal continuation could only have mismatching targets (as a necessary design limitation, see the Methods section for details). Thus, the critical conditions in Experiment 2 for testing prediction across modalities involved the three comparable types of mismatching trials, as illustrated in Figure 3. It is critical to note that the comparison of greatest interest in this experiment is between the discontinuous and the cross-modal continuous conditions, both of which involve an identical video splice (or audio splice) between context and target fragments. Because the discontinuity from context to target portions of the sentences is identical in these cases, it cannot lead to differences in attentional capture at the splice point.

**Figure 4:**
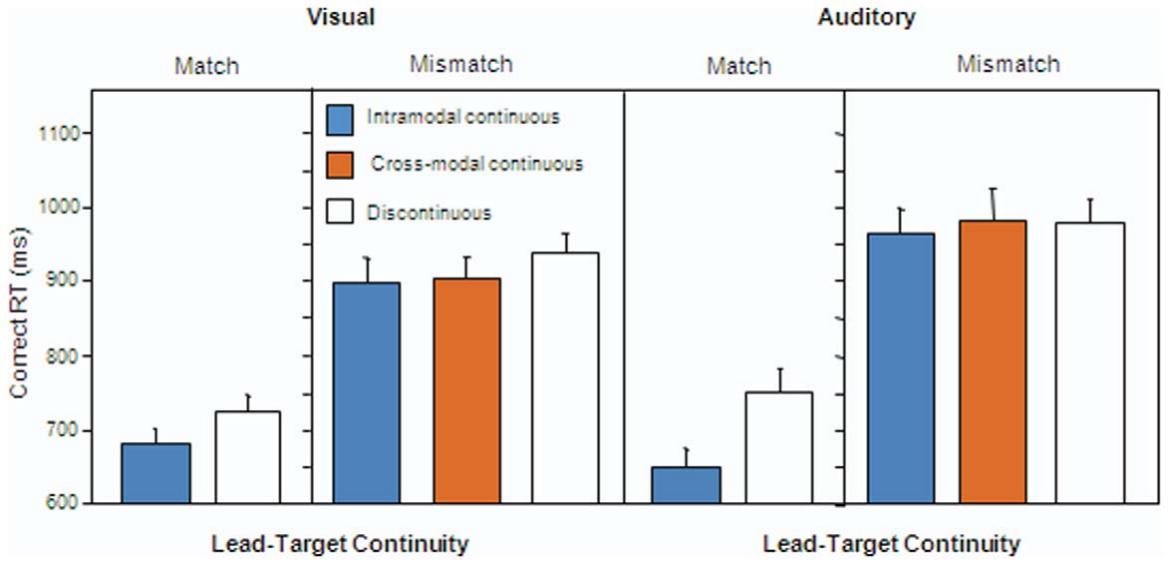
Mean correct RT (in milliseconds) in Experiment 2. Visual (left panel) and auditory (right panel) versions. Error bars represent one standard error of the mean.

Figure 4 shows the mean correct response times in Experiment 2. In the visual version (left side), participants were able to detect audiovisual matches more rapidly following a continuous versus a discontinuous leading context. They were also able to detect mismatches more rapidly following both an intra-modal and a cross-modal continuation, as compared to the discontinuous condition. The auditory version (right side) revealed the same pattern of results, with one exception. Although the data showed an advantage for intra-modal continuity over discontinuity on matching trials and mismatching trials, there was no evidence of a benefit when the continuity was cross-modal.

An ANOVA including the factors of context continuity (intra-modal continuous vs. discontinuous) and target congruence (match vs. mismatch), revealed faster responses when the context was continuous intramodally than discontinuous (visual: F (1,15) = 15.3, p<0.05; auditory: F(1,15) = 26.99, p<0.05), and when the target fragment was matching rather than mismatching (visual: F(1,15) = 186.16; p<0.05; auditory: F(1,15) = 115.63; p<0.05). This result supports the within modality continuous context advantage found in Experiment 1, with a different baseline (discontinuous context, rather than no context). The interaction between context and congruence was not significant in the visual version, F(1,15) = 2.38, p = 0.14, but it was in the auditory version, F(1,15) = 26.19, p<0.05.

A second ANOVA included all three types of context continuity (but only mismatching trials, given the design constrain discussed in the Methods section). This was the critical analysis to test the hypothesis of cross-modal prediction. The main effect of type of context was significant in the visual version, F(2,30) = 7.72, p<0.01, but not in the auditory version, F(2,30) = 0.412, p = 0.66. Follow-up tests in the visual version showed that RTs in both the intra- and cross-modal continuation conditions were faster than the discontinuous condition (t(15) = 3.24, p = 0.05; t(15) = 3.26, p<0.05, respectively), and not different from one another, t(15) = 0.83, p = 0.41. Equivalent tests in the auditory version failed to reach significance, all |t|<1. Overall, participants were slightly faster responding after a visual leading context than after an auditory context, but the difference was not significant (t(30) = 1.38; p = 0.17).

Like in Experiment 1, response accuracy was high (visual = 90%, auditory = 84%). In the visual version, intra-modal continuation performance (*d′* = 2.84) was superior to that of discontinuous (*d′* = 2.60), (t(15) = 2.76, p<0.05), and there were no significant differences between cross-modal continuation (*d′* = 2.58) and discontinuous, t(15) = 0.24, p = 0.81. In the auditory version, there were no significant differences, intra-modal continuous, *d′* = 1.88; discontinuous, *d′* = 1.92 and cross-modal, *d′* = 1.86, all |t|<1. The criterion was not significantly different from zero in any of the two versions (visual version: intra-modal continuous, *C* = 0.03, t(15) = 0.63, p = 0.53); cross-modal continuous, *C* = −0.10, t(15) = −1.26, p = 0.22; discontinuous, *C* = −0.09, t(15) = −1.28, p = 0.21. Auditory version: intra-modal continuous, *C* = 0.02, t(15) = 0.35, p = 0.73; cross-modal continuous, *C* = 0.01, t(15) = 0.118, p = 0.90; discontinuous, *C* = 0.04, t(15) = 0.54, p = 0.59)), indicating the absence of bias towards any kind of response.

## Discussion

This study offers behavioral evidence that listeners can use speech information on-line to constrain the interpretation of the subsequent signal within and across sensory modalities, thereby benefiting performance in an audiovisual speech matching task. When the leading context fragment (auditory or visual) was continuous within the same modality in the audiovisual target fragment, there was a reduction in response time for the detection of audiovisual mismatch (Experiments 1 and 2). However, when the context and target fragments were continuous across different modalities, only visual continuity into auditory channel (but not the reverse) produced a benefit. We interpret these results as indicating that at least under some conditions, immediately preceding speech context can be used to form predictions about the upcoming input, facilitating the detection of a mismatch between audio and visual channels. And in the case of visual to auditory transfer, the information can even be transferred within the time limits of the modality switch.

These results can be readily interpreted within a predictive coding framework. In these models, speech information at various levels of processing (i.e., semantic, syntactic, phonological) is extracted from the signal and used to activate hypotheses at levels above (feedforward processing) and below (feedback processing). Such an arrangement allows the system to constantly generate probabilistic hypotheses about the upcoming signal to constrain the interpretation of the incoming input on-line.

Unlike the visual context, the auditory context fragment was clearly comprehensible for the observers. Thus, the beneficial effect of the auditory context during Experiment 1 may not be too surprising, as it allows for the possibility of predictions to be formed at higher levels (semantic, syntactic) as well as lower ones (phonological, articulatory). As such, the benefit of context in the auditory version is consistent with previous ERP evidences for auditory-based predictions being used on-line in the comprehension of spoken language [27]. It may be also related to previous demonstrations of on-line predictions being used in the comprehension of written language (e.g., [28], [29]).

However, to our knowledge, this study provides the first demonstration that prior visual speech-reading information can be used to benefit speech processing in a similar way. One important difference, however, is that the visual speech signal provided very little information to our participants, who are not trained lip-readers, at the levels of syntax and semantics [4], [36]. Therefore, we believe that in the audiovisual matching task used in our experiments, the phonological or pre-phonological levels are the most likely used for cross-modal transfer from vision to audition. For instance, phonology is claimed to be amongst the earliest representational levels at which auditory and visual aspects of speech can be encoded in a common format (e.g., [30], [31]). As reviewed in the Introduction section, phonology is likely the level where facial articulatory movements correspond most closely to acoustic signals, perhaps based on the link they both are supposed to have with the articulatory representations used in speech production [22], [24], [30], [31], [32].

To support this interpretation, we estimated the amount of semantic and syntactic information that could be extracted from the visual leading context in our stimuli. In order to do it, we tested twelve new participants with thirty-nine of the sentences used in Experiments 1 and 2, presented only visually. Participants were asked to report, after watching each sentence, the words that they had been able to recognize. We scored the proportion of content words correctly reported (i.e., nouns, verbs and adjectives but not functional words such as articles or prepositions). The mean percentage of correctly reported words was 3.2%, which supports our claim that information at lexical or higher levels could be hardly extracted from the visual context. It is more likely that the information extracted and used in cross-modal transfer is of a pre-lexical nature (phonological, pre-phonological or perhaps even prosodic) rather than semantic.

The distinction between the possible role of phonological and pre-phonological levels in our results is, at this point, difficult. Some theories of audiovisual fusion claim for the existence of a common format at an early, pre-phonological level of representation [37], [38]. We cannot rule out or confirm the possibility that the prediction effects will be based on such levels of representation with our current evidence. A potential way to address the role of phonological vs. pre-phonological representations would be to test for prediction effects in an unknown language. If prediction effects equivalent to those seen here happen at a phonological level rather than in a pre-phonological one, then some minimal degree of phonological knowledge about the language will be necessary for cross-modal transfer to occur.

Our data imply that visual speech information can be used to constrain processing of subsequent auditory information, through a real-time intra-modal transfer as well as a cross-modal transfer of information. This cross-modal benefit is, however, unidirectional from visual to auditory, but not vice-versa. Why the cross-modal transfer was asymmetric, showing benefits of leading visual information on audition, but not the reverse? Our interpretation is that this is consistent with bio-mechanical constraints on language production, whereby the visual information available to an observer precedes in time the corresponding acoustic information [34]. It also fits well with previous ERP findings in which auditory evoked potentials occur earlier when correlated visual information is present [26], [35]. However, an alternative explanation for the present asymmetry in cross-modal effects is that speech comprehension based on the visual channel alone is so much more difficult than when based on the auditory channel alone. As such, the visual leading context may prompt participants to try to actively simulate the sounds based on the facial gestures. In contrast, merely listening to an auditory leading context would not prompt the same degree of active involvement in the task, given that comprehension is easy. To test this hypothesis we conducted a control experiment, identical to the auditory version of Experiment 2, with the exception that a simultaneous noise mask was added to the auditory channel (Signal to Noise Ratio = −5 dB) in order to render it barely intelligible. Despite the increased effort now required to understand the auditory channel, the correct RT data replicated the main result of the auditory version in Experiment 2 (RTs in the cross-modal continuous condition were not significantly different from the discontinuous one (average RTs = 1156.76 ms vs. 1143.78 ms; t(19) = 1.22, p = 0.23). This result rules out the *difficulty* hypothesis, although it must be admitted that the asymmetry in our results could be due to strategic differences resulting from extended experience with audio emulation from lip-reading but not visual emulation from audio perception, making the cross-modal transfer more likely from vision to audio than in the opposite direction. Our data does not allow us to resolve this question at present.

Interestingly, in Experiment 1 (auditory and visual version) the benefits of prediction tended to be larger when the task demanded the detection of audiovisual mismatch rather than a match, whereas matching trials showed a benefit of continuity only in Experiment 2. This is in accord with a recent suggestion of an important processing difference on audiovisual match versus mismatch signals [39]. Arnal et al. proposed that when sensory modalities match, they engage preferentially direct connections between visual and auditory areas. In contrast, mismatching information across modalities engages a slower, more indirect network, whereby visual input is integrated and compared with the auditory input via association areas (i.e., the Superior Temporal Sulcus, STS). As such, the process of detecting match in the present study may have occurred too rapidly to be indexed by our response time measure in Experiment 1. The quicker responses to matching trials, as compared to mismatching ones, together with the significant bias to respond ‘match’ in several of the conditions tested in Experiment 1 (informative context (visual version), *C* = −0.04, t(15) = 0.62, p = 0.54; *C* = −0.19, t(17) = −3.87, p<0.01(auditory); no context, *C* = −0.37, t(15) = −5.35, p<0.01(visual); *C* = −0.38, t(17) = −5.61, p<0.01(auditory)), may reflect a strategy in which participants would default to a matching response a priori. From this perspective, checking for disconfirmation (mismatching responses), would take longer than checking for a confirmation (matching responses). The significant bias toward matching responses in Experiment 1 would support this hypothesis. Note, however, that in Experiment 2, precisely where the on-line cross-modal transfer was shown, there were no significant criterion shifts. Therefore, this particular strategy cannot be the only cause of the RT pattern reported here.

The neural mechanism that mediates this improvement of audiovisual processing following a visual context still remains unknown. We could speculate about the involvement of the mirror neuron system, as suggested by some authors. According to the model proposed by Skipper et al. [24], for example, while perceiving visual information, the motor system is engaged in comparing a hypothesis based on previous experience (forward model) and the perceived information. This makes possible to speed up processing about incoming information that matches expectations.

In conclusion, the present study documents an important case of on-line cross-modal transfer of information in speech perception. Specifically, it demonstrates that visual speech signal in a sentence can facilitate the quick extraction of sufficient information for the detection of a match or mismatch in a subsequent audiovisual portion of the sentence. Our results support that on-line speech perception benefits from a leading visual information, that can be used both to constrain the interpretation of subsequent visual (intra-modal) and auditory (cross-modal) processing. In the case of leading auditory information, the benefit occurs only within the same sensory modality. These results may reflect the well known precedence of visual to acoustic consequences of articulation. We contend that this predictive ability may play a facilitatory role in everyday communication, enabling phonological predictions, based on visual cues, of what we are about to hear.

## Methods

### Experiment 1: Benefits of prior visual and auditory information

#### Participants

Data from 34 native Spanish speakers (10 males, mean age 23.4 years) were included in Experiment 1. Data from eight participants who failed to meet a performance criterion of 65% accuracy in the audiovisual matching task were not included, so that their data did not alter our conclusions. All participants reported normal audition and normal or corrected-to-normal vision, and were naive to the purpose of the experiment. The protocol was run under the approval of the University of Barcelona ethics committee, and all participants gave written informed consent. Sixteen participants were assigned to the visual leading context version; 18 to the auditory leading context version.

#### Materials and procedure

The stimuli consisted of high resolution audiovisual recordings of a male speaking fifty-two complete sentences in Spanish, as indicated in the Appendix S1. Each sentence was edited with Adobe Premiere Pro 1.5, to last 2400, 2600, 2800 and 3000 milliseconds, and included a 560 ms linear fade-in ramp and a 360 ms linear fade-out ramp. Participants viewed the video recordings from a distance of 60 cm on a 17″ CRT computer monitor that showed the full face of the speaker face in the center of the screen. The audio channel was played through two loudspeakers located on each side of the monitor, at a comfortable listening intensity of 65 dB SPL. A program using DMDX software [40] was used to organize the randomization, presentation and timing of the experiments.

Temporal uncertainty was created by sampling randomly and equiprobably among four leading context durations (1600, 1800, 2000 and 2200 ms), prior to the presentation of the 800 ms target fragment. Trials began with a central fixation circle (0.8° visual angle, 500 ms), followed by the presentation of a sentence context (1600–2200 ms) plus target (800 ms). Following each response or time-out (1800 ms deadline) the screen blanked for 800 ms before the next trial began. To confer ecological validity to our design, we left at random the level of discriminability of the particular articulatory gesture in which each of the sentences change form context to target. We just avoided that the transition would occur during a speech (silent) pause in the sentence.

Participants judged, as quickly and accurately as possible, whether the target fragment of the sentences had matching or mismatching audiovisual channels. Responses were made with the index and middle fingers on two neighboring keys, with the assignment of finger to response counterbalanced across subjects. The target fragment consisted of the final 800 ms of each sentence, and it always included both audio and visual channels. To create mismatching targets from these recordings, the audio (or visual, depending on the version) channel of the original fragment was randomly replaced with that of another sentence.

In order to test the effect that both modalities could have over the audiovisual matching task, we ran two different versions of Experiment 1. In one version, we presented an auditory leading context, and in the other version, we presented a visual leading context. In each of the two versions, there were four different types of trials, formed from the orthogonal combination of whether the leading context was a sentence fragment or not (leading context, no context) and whether the audiovisual channels in the target fragment were matching or mismatching. The leading context was always either the original audio or the original visual fragment of the sentence that preceded the target fragment, and thus it continued from the context through the target fragment. The channel that was not informative during this unimodal leading context was replaced. The replacement of the auditory channel was a sequence of rhythmic beats (300 Hz tones, 120 ms duration each, presented at 5 Hz, as shown in Figure 1), that was comparable to the rhythm of speech, and the visual channel was replaced with a still face of the speaker. For the no context conditions, used as the baseline, a still frame of the speaker's face was combined with rhythmic beats. It is important to note that the leading context manipulation (present or absent) was orthogonal with respect to the task and response set, which was whether the audiovisual channels were matching or non-matching. Each participant responded to a total of 208 trials in either the visual or the auditory version, with each of the 52 original sentences edited to create the 2×2 design: leading context vs. no context, and matching vs. mismatching target. Only in two of the four times that each sentence was presented to each participant, it was shown on its complete form, including context, making any possibility of learning very unlikely. These sentences were sampled randomly without replacement for each participant, with context duration varying randomly and equiprobably amongst the four possible durations (1600 to 2200). Participants practiced on a subset of 20 sentences prior to testing. Each experimental session lasted approximately 30 min.

### Experiment 2: Cross-modal vs. intra-modal predictions

#### Participants

A different group of participants, formed by 32 native Spanish speakers (10 male, mean age 23.1 years) participated in Experiment 2. Data from 17 additional participants who failed to meet the 65% performance criterion were not included, so that their data did not alter our conclusions. Sixteen participants were assigned to the visual leading context version; 16 to the auditory leading context version.

#### Materials and procedure

Forty audiovisual sentences similar to those used in Experiment 1 were selected. As in Experiment 1, we created two versions of the experiment, one to test for visual-to-auditory prediction (called *visual version* for simplicity) and one to test for auditory-to-visual prediction (called *auditory version*). As in Experiment 1, participants judged if the target fragment was audio-visually matching or mismatching.

The critical comparisons in this experiment involved the three audiovisual mismatching target conditions illustrated in Figure 3. The condition called *intra-modal continuous* was identical to the context condition of Experiment 1, in that the context channel was continuous with the same channel in the target fragment. In the new condition called *cross-modal continuous*, the leading context channel was continuous with the alternative modality channel in the target fragment. Finally, the *discontinuous* condition served as a comparison for both of these continuous conditions, in that it required the same response (a mismatch judgment), but the leading context provided no information about the message in the target clip (since it belonged to a different sentence).

Each participant was tested in a total of 200 trials, distributed in 5 equivalent blocks of 40 trials in which each trial type was equiprobable. Only in the two continuous conditions participants were presented with the complete form of the sentences, to avoid any possibility of learning. The experimental session lasted about 30 min.

## Supporting Information

Appendix S1 Spanish sentences and their English translation.
(DOC)
