# Supplementary material for: Cross-Modal Prediction in Speech Perception
Source: PLoS One. 2011 Oct 5;6(10):e25198. doi: 10.1371/journal.pone.0025198 (PMC3187777; doi:10.1371/journal.pone.0025198)
Supplement: Appendix S1 — Spanish sentences and their English translation. (DOC) [file pone.0025198.s001.doc]

1. Pensando que así el queso se mantendría más fresco.

*Thinking that cheese would keep fresher this way.*

1. Entró en un castillo y vio a un malvado gigante que tenía un caballo blanco.

*He came into a castle and saw an evil giant holding a white horse.*

1. Con un pico tan largo no podía coger los trozos de comida más grandes.

*It was hard for him to pick the biggest pieces of food with such a long beak.*

1. Mi hermano y yo estábamos ocultos en una extraña terraza de laureles.

*My brother and me were hidden in a strange terrace of bay leaves.*

1. Un rato después de la visita yo ya había olvidado esa cara redonda y llena.

*Sometime after the visit I have already forgotten that round and fat face.*

| 1. El héroe del cuento fabricaba una máquina para producir espadas.   *The hero of the tale was constructing a machine to make swords.*   1. Le mostré la estatua china de piedra verde que yo había comprado esa misma mañana.   *I showed him the Chinese statue made of green stone that I bought that very same morning.* |
| --- |

1. Salió para buscar un bar en el que hicieran café de calidad.

*He went out to find a bar in which good quality coffee was made.*

1. Me tapé con la manta, me tumbé en el sofá de casa.

*I covered myself with the blanket, I lied down in my home’s sofa.*

1. Enseguida se levanta, guarda la rosa entre sus manos.

*He quickly stands up, keeping the rose into his hands.*

1. La fruta relucía en la mesa, parecía de mentira.

*The fruit was shining on the table, it looked like it was fake.*

1. Los dos viejos amigos se acercaron cada uno a un espejo.

*Each of the two old friends walked towards a mirror.*

1. No daba nunca las gracias y nunca levantaba la vista para saber quién era el donante.

*He never neither said thanks nor lift his gaze to know who the donor was.*

1. En las afueras de la ciudad, escondida entre los árboles, se encontraba una pequeña casita blanca.

*In the suburbs of the city, hidden in the trees, there was a little white house.*

1. Un día mientras el hombre se encontraba ya trabajando en el pueblo.

*One day, while the man was already working in the village.*

1. Los cristales estaban tan limpios que reflejaban la luz del sol.

*The glasses were so clean that they reflected the sunlight.*

1. Los libros estaban clasificados en diferentes temas.

*Books were classified in different topics.*

1. Como el ordenador no funcionaba llamaron a un técnico.

*As the computer was not working, they phoned a technician.*

1. La gente tenía que encender sus chimeneas casi toda la noche.

*People had to turn on the chimneys almost during all night.*

1. Ese hombre era conocido como el cómico más divertido del mundo.

*That man was known as the funniest comedian worldwide.*

1. El guitarrista era feliz sobre el escenario, tocando hasta la madrugada.

*The guitar player was happy on stage, playing until dawn.*

1. Después de caminar todo el día por un camino muy difícil al fin vieron las primeras luces del pueblo.

*After walking all day through a very hard path, they finally saw the first lights of the village.*

1. Cuando llegaron a la casa estaba muy sucia.

*When they arrived to the house, it was very dirty*

1. Hay tan pocas cosas por las que estar contento y feliz.

*There are a very few things deserving you to be merry and happy.*

1. Pasó el verano y vino el otoño y el jardín continuó dando flores.

*Summer went past, fall arrived, and the garden kept on bringing flowers.*

1. Los pocos que quedaban empezaron a reconstruir el castillo.

*The few people left started to re-build the castle.*

1. Su gran ilusión era salir de su casa durante el día.

*His greatest illusion was to get out of the house during the daytime.*

1. En ocasiones, a uno le hace falta vivir una tragedia para volver a poner las cosas en perspectiva.

*Sometimes, it is necessary to go through a tragedy to be able to put things again into perspective.*

1. La oficina estaba totalmente vacía cuando ellos se fueron.

*The office was completely empty when they left.*

1. El hombre que le tenía que vender la bicicleta no cogía el teléfono.

*The man who had to sell the bicycle didn’t answer the phone.*

1. El agua de la lluvia, que ha entrado sin parar, se ha colado hasta mis rodillas.

*Rain’s water, which did not stop coming in, has already reached my knees.*

1. Tenían la televisión a un volumen tan alto que no se podían entender.

*The volume of the television was so high that they could not understand each other.*

1. Todos los relojes se pararon a la misma hora.

*All clocks stopped at the same time.*

1. El sol se filtraba entre los rascacielos del centro*.*

*Sun filtered through skycrapers in the city center.*

1. Acostado en la cama de matrimonio, un hombre con los ojos húmedos leía una carta que le trajo el correo.

*Lying in a double bed, a man with wet eyes was reading the letter that the postman brought.*

1. La policía llegó a la manifestación de estudiantes tan rápido como pudo.

*Police arrived to the student protest as soon as they could.*

1. El bosque estaba muy quieto cuando los hombres llegaron.

*The forest was very quiet when the men arrived.*

1. El hombre de blanco le miró desde lejos, sonriendo.

*The man dressed in white watched him from the distance, smiling.*

1. Los patos se acercaron a ellos, esperando a que les tiraran un poco de comida.

*Ducks approached them, waiting for them to throw some food.*

1. Era inútil abrir los ojos y mirar en todas direcciones.

*It was useless to open the eyes and to look everywhere.*

1. Pensó que debía haber gritado, en medio de su sueño.

He thought he should have shout, while dreaming.

1. Susana se quedó delgada, no sabía qué comer porque ya nada le gustaba.

*Susana ended up being very thin, she did not know what to eat as she did not like anything now.*

1. Una niña se aproximó a la tienda y apretó la naricita contra el vidrio del escaparate.

*The little girl approached to the shop and she pushed her nose against the shop window.*

1. Un perro corría por toda la casa con un pañuelo atado en la cola.

*The dog run around all the house with a shawl tied in the tail.*

1. Hoy por la mañana hemos ido a dar una vuelta por el campo.

*Today in the morning, we have gone for a walk in the countryside*.

1. Pasaron algunas horas y el sueño me venció.

*Some hours passed and sleep beat me.*

1. Al segundo día escalamos hasta la montaña más alta de esa región.

*The second day we climbed to the highest mountain in that region.*

49. Con estos pensamientos el viaje de dos horas pasa como un suspiro.

*Having those thoughts, the two hours trip passed very quickly.*

1. Se dio una ducha rápida y se preparó un café negro.

*He took a quick shower and he made himself a black coffee.*

1. La pulida superficie del espejo le devolvió una imagen clara.

*The clean surface of the mirror gave him back a clear image.*

1. Para poder pasarles la pensión empecé a trabajar turnos dobles en la fábrica.

*To be able to pay the rent, I started to work on double shift in the factory.*
